# Supplementary material for: Evolution of Bacterial Persistence to Antibiotics during a 50,000-Generation Experiment in an Antibiotic-Free Environment
Source: Antibiotics (Basel). 2022 Mar 27;11(4):451. doi: 10.3390/antibiotics11040451 (PMC9028194; doi:10.3390/antibiotics11040451)
Supplement: Supplementary file 1 [file antibiotics-11-00451-s001.zip › antibiotics-1634163-supplementary/Supplementary materials.pdf]

Table des matières

Table S1: Pairwise comparisons of the abundance of ciprofloxacin persister cells in the evolved clones sampled from each of the 12 LTEE populations at 50,000 generations. 2

Figure S1: Effect of antibiotics on growth rates. 3

Supplementary method 1: Heuristic selection of the exponential growth phase 3

Figure S2: Variability in background OD for the wells of a representative microplate. 4

Figure S3: Illustration of the detection of the exponential phase in the growth curve. 5

Table S1: Pairwise comparisons of the abundance of ciprofloxacin persister cells in the evolved clones sampled from each of the 12 LTEE populations at 50,000 generations.

**Ara**	-2L	-2S	-3	-4	-5	-6	+2	+3	+4	+5	+6
-2L		ln(FC) = 5.81<br>p < 0.001 ***		ln(FC) = 5.58<br>p = 0.016 *			ln(FC) = 5.51<br>p = 0.013 *				
-2S											
-3											
-4											
-5											
-6											
+2											
+3											
+4		ln(FC) = 9.57<br>p < 0.001 ***	ln(FC) = 6.76 ;<br>p < 0.001 ***	ln(FC) = 9.34<br>p < 0.001 ***	ln(FC) = 6.23<br>p = 0.009 **	ln(FC) = 7.83<br>p < 0.001 ***	ln(FC) = 9.27<br>p < 0.001 ***	ln(FC) = 6.39<br>p = 0.002 **			
+5		ln(FC) = 6.6<br>p = 0.001 **		ln(FC) = 6.37<br>p = 0.036 *			ln(FC) = 6.29<br>p = 0.034 *				
+6		ln(FC) = 5.71<br>p < 0.001 ***		ln(FC) = 5.48<br>p = 0.03 *			ln(FC) = 5.4<br>p = 0.028 *				

These comparisons are based on the coefficients of the model in Table 1. The ln(FC) values give the logarithm of the fold change of the abundance of persister cells. This fold change is defined as the abundance in the clone in the row over the abundance in the clone in the column. In non-empty cells, persistence is significantly higher in the clone in the row than in the clone in the column. The significance of this fold change was assessed with the R package `multcomp`. For readability, the clones sampled from populations Ara–1 and Ara+1 were removed because they do not significantly differ from any other clones, and only significant results are shown. For the full results, see tables S2 and S3. Significance codes: 0 '***' 0.001 '**' 0.01 '*' 0.05 '.' 0.1 ' ' 1

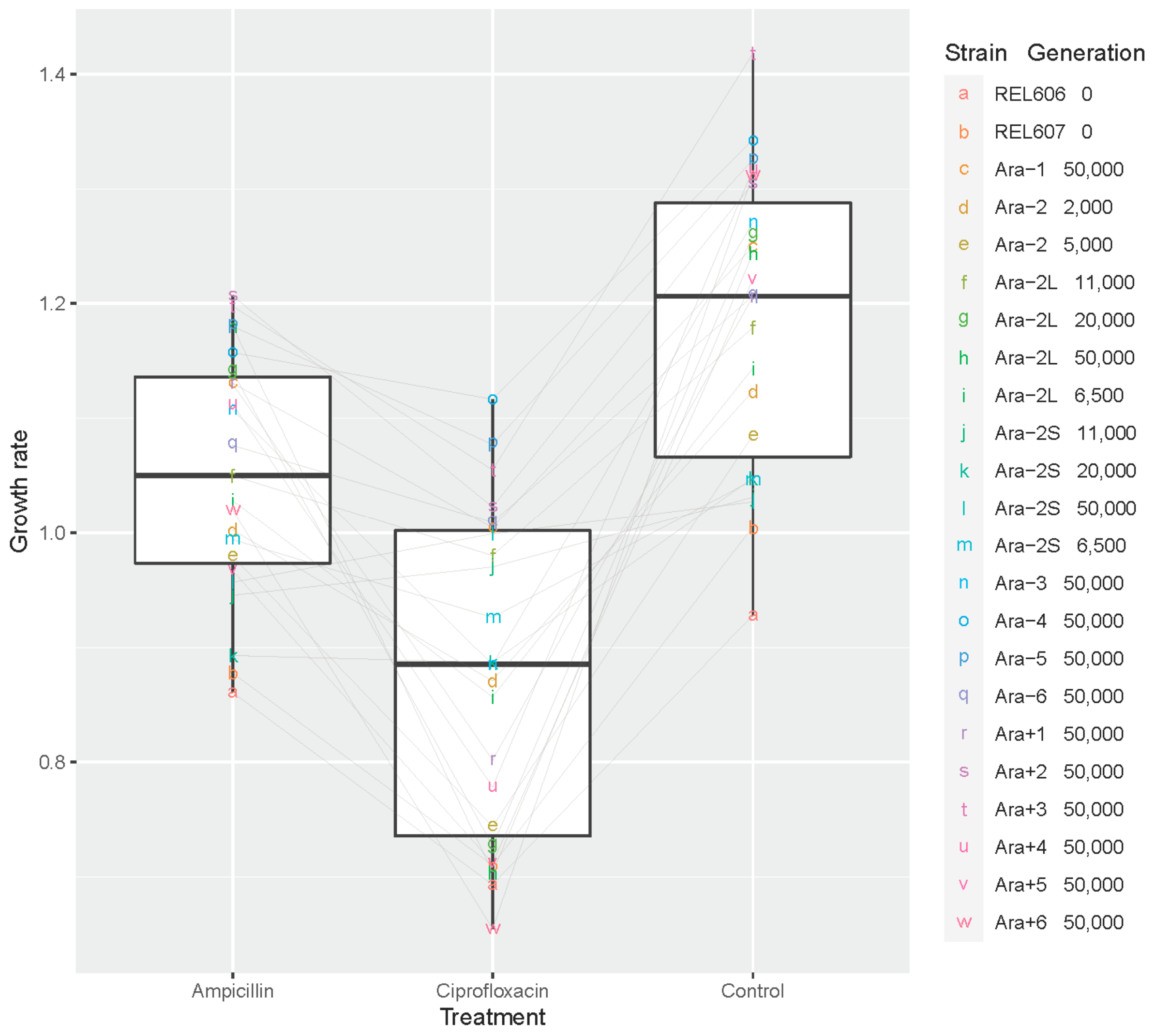

Figure S1: Effect of antibiotics on growth rates.

Each combination of letter and color corresponds to one clone.

Lines link each individual clone in each treatment.

In the boxplots: the central line give the median, the extremities the first and third quartiles, and the vertical bars the minimal and maximal values.

Supplementary method 1: Heuristic selection of the exponential growth phase

We detected the exponential growth phase in two steps that were implemented by the R function we developed: "*SimpleExponentialGrowthFit*" (file *SimpleExponentialGrowthFit.R)*. The rationale is similar to the approach implemented by the R function '*fit_easylinear*' (details and comparison below).

In the **first** step, we identified the part of the growth curve with the highest exponential link between OD_{600} and time by using a sliding window approach. While '*fit_easylinear*' implicitly assumes that the background was removed by the user, our function estimates the background separately for each growth curve. This is important since the background varied a lot within the same plate (Figure S2).

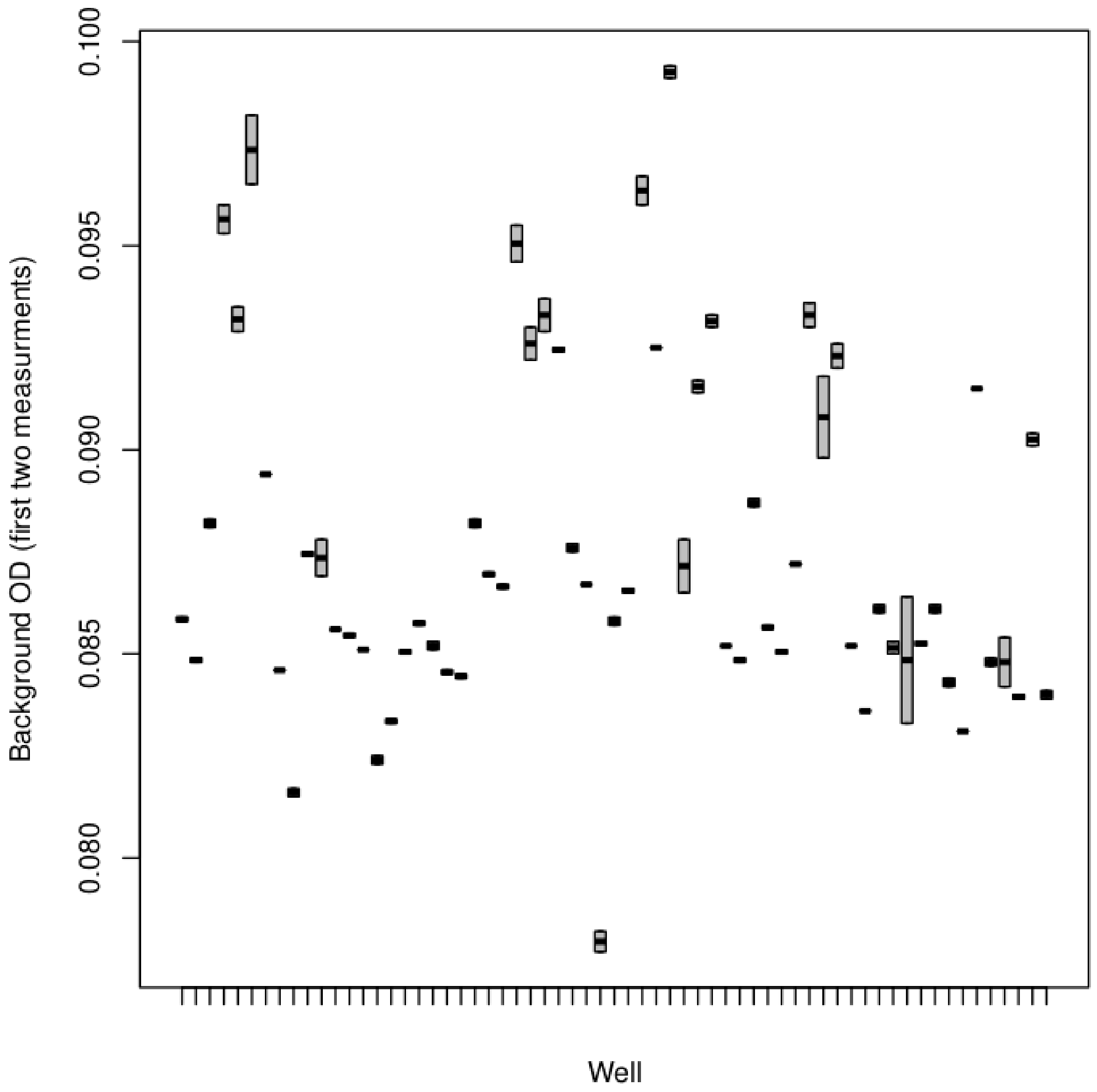

Figure S2: Variability in background OD for the wells of a representative microplate.

To consider the entire exponential growth phase, the **second** step aimed at extending the span of the window with the highest exponential link between OD_{600} and time that was detected during the first step.

We detected and fitted exponential growth for each growth curve as follow.

Step 1: identifying the window with the highest exponential link between OD_{600} and time.

For each sliding window of 10 time points (2.5h) we fitted and scored the linear model $log_2(OD - Bg)_i = GrowthRate \times time_i + OD_{init} + \varepsilon_i$, where:

- Bg is the background OD that is specific to the sliding window. It is estimated as the median OD in the first ¾ of the dots before the start of the current sliding window (*e.g.*, in Figure S3).
- $time_i$ is the explanatory variable.
- $GrowthRate$ is the slope associated with this variable: the time needed for $OD - Bg$ to be multiplied by two is $1/GrowthRate$.

To see this, define two time points a and b, such as $OD_b - Bg = 2 \times OD_a - Bg$ $\Leftrightarrow\ log_2(OD_b - Bg) = log_2(2) + log_2(OD_a - Bg) = 1 + log_2(OD_a - Bg)$.
This equation can be rewritten with the right side of the model:
$GrowthRate \times time_b + OD_{init} = 1 + GrowthRate \times time_a + OD_{init}$
$\Leftrightarrow GrowthRate \times (time_b - time_a) = 1 \Leftrightarrow time_b - time_a = 1/GrowthRate$.

- OD_{init} is the intercept that corresponds to the initial OD. Indeed, at $time_1 = 0$, we have $OD_0 - Bg = 2^{OD_{init}}$. In the absence of lag phase, this value would correspond to $a \times N_0$ where N_0 is the initial number of cells, and a describes the relationship between OD and N: $OD - Bg = a \times N$. However, since a lag phase is present in most cases, $2^{OD_{init}}$ is positively associated with N_0 and negatively to the lag phase.

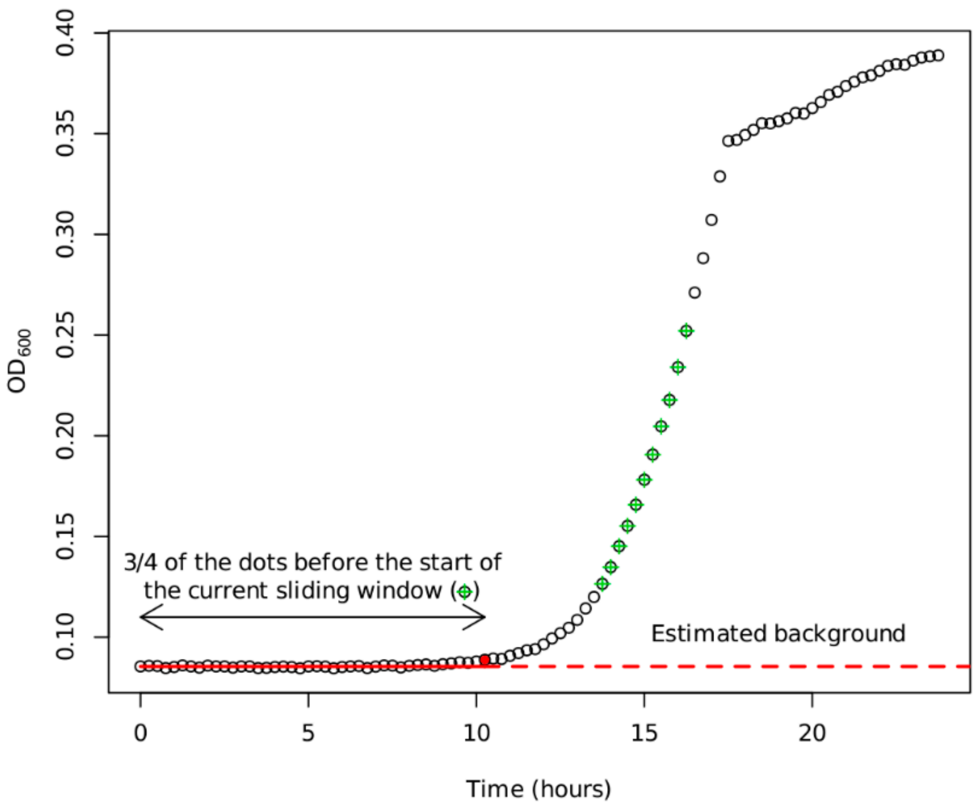

Figure S3: Illustration of the detection of the exponential phase in the growth curve.

We scored the model of each sliding window with the formula:
$GrowthRate \times ln(standard\ deviation\ of\ the\ OD\ in\ the\ conscidered\ sliding\ window + 1) \times R^2$
and selected the sliding window with the highest score.

In this formula, the ln of the OD standard deviation avoids the window being selected earlier than the exponential growth, *i.e.*, during the lag phase, when growth revealed to be noisy.

Step 2: extending the span of the window.

We extended the window span for it to include the entire exponential phase. We chose the span of the window that maximized $\overline{-log\left(\left|\widehat{OD} - OD\right|\right)} \times R^2$ where $\widehat{OD}$ is the predicted OD by extrapolating to the entire growth curve the model adjusted to the selected window. The use of the log of the absolute value of the error allowed to set a low weight to large errors that corresponded to the growth period outside the exponential growth phase.
