# Supplementary material for: Evolution of Bacterial Persistence to Antibiotics during a 50,000-Generation Experiment in an Antibiotic-Free Environment
Source: Antibiotics (Basel). 2022 Mar 27;11(4):451. doi: 10.3390/antibiotics11040451 (PMC9028194; doi:10.3390/antibiotics11040451)
Supplement: Supplementary file 1 [file antibiotics-11-00451-s001.zip › antibiotics-1634163-supplementary/Supplementary material 1- Heuristic detection of the exponential growth phase.zip!/Supplementary material 1- Heuristic detection of the exponential growth phase/0_Description of the heuristic selection of the exponential growth phase.pdf]

Heuristic selection of the exponential growth phase

We detected the exponential growth phase in two steps that were implemented by the R function "*SimpleExponentialGrowthFit*" that we developed (file *SimpleExponentialGrowthFit.R)*. The rationale is similar to the approach implemented by the R function '*fit_easylinear*' (details and comparison below).

In the **first** step, we identified the part of the growth curve with the highest exponential link between OD_{600} and time by using a sliding window approach. While '*fit_easylinear*' implicitly assumes that the background was removed by the user, our function estimates the background separately for each growth curve. This is important since the background varied a lot within the same plate (Figure S2). To consider the entire exponential growth phase, the **second** step aimed at extending the span of the window with the highest exponential link between OD_{600} and time that was detected during the first step.

We detected and fitted exponential growth for each growth curve as follow.

Step 1: identifying the window with the highest exponential link between OD_{600} and time.

For each sliding window of 10 time points (2.5h) we fitted and scored the linear model $log_2(OD - Bg)_i = GrowthRate \times time_i + OD_{init} + \varepsilon_i$, where:

- Bg is the background OD that is specific to the sliding window. It is estimated as the median OD in the first ¾ of the dots before the start of the current sliding window (*e.g.*, in Figure S3).
- $time_i$ is the explanatory variable.
- $GrowthRate$ is the slope associated with this variable. Indeed, the time needed for $OD - Bg$ to be multiplied by two is $1/GrowthRate$. To see this, define two time points a and b, such as
  $OD_b - Bg = 2 \times OD_a - Bg$
  $\Leftrightarrow\ log_2(OD_b - Bg) = log_2(2) + log_2(OD_a - Bg) = 1 + log_2(OD_a - Bg).$
  This equation can be rewritten with the right side of the model:
  $GrowthRate \times time_b + OD_{init} = 1 + GrowthRate \times time_a + OD_{init}$
  $\Leftrightarrow GrowthRate \times (time_b - time_a) = 1 \Leftrightarrow time_b - time_a = 1/GrowthRate.$
- OD_{init} is the intercept that corresponds to the initial OD. Indeed, at $time_1 = 0$, we have $OD_0 - Bg = 2^{OD_{init}}$. In the absence of lag phase, this value would correspond to $a \times N_0$ where N_0 is the initial number of cells, and a describes the relationship between OD and N: $OD - Bg = a \times N$. However, since a lag phase is present in most cases, $2^{OD_{init}}$ is positively associated with N_0 and negatively to the lag phase.

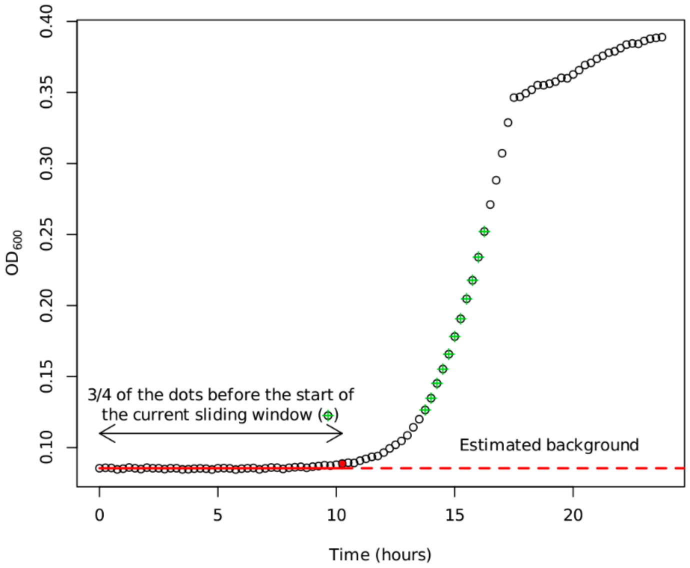

Figure S3: Illustration of the detection of the exponential phase in the growth curve.

We scored the model of each sliding window with the formula:

$$GrowthRate \times ln(standard\ deviation\ of\ the\ OD\ in\ the\ conscidered\ sliding\ window + 1) \times R^2$$

and selected the sliding window with the highest score.

In this formula, the ln of the OD standard deviation avoids the window being selected earlier than the exponential growth, *i.e.*, during the lag phase, when growth revealed to be noisy.

Step 2: extending the span of the window.

We extended the window span for it to include the entire exponential phase. We chose the span of the window that maximized $\overline{-log\left(\left|\widehat{OD} - OD\right|\right)} \times R^2$ where $\widehat{OD}$ is the predicted OD by extrapolating to the entire growth curve the model adjusted to the selected window. The use of the log of the absolute value of the error allowed to set a low weight to large errors that corresponded to the growth period outside the exponential growth phase.
