# Supplementary material for: Evolution of Bacterial Persistence to Antibiotics during a 50,000-Generation Experiment in an Antibiotic-Free Environment
Source: Antibiotics (Basel). 2022 Mar 27;11(4):451. doi: 10.3390/antibiotics11040451 (PMC9028194; doi:10.3390/antibiotics11040451)
Supplement: Supplementary file 1 [file antibiotics-11-00451-s001.zip › antibiotics-1634163-supplementary/Supplementary material 1- Heuristic detection of the exponential growth phase.zip!/Supplementary material 1- Heuristic detection of the exponential growth phase/Figure explaining detection growth curve.pdf]

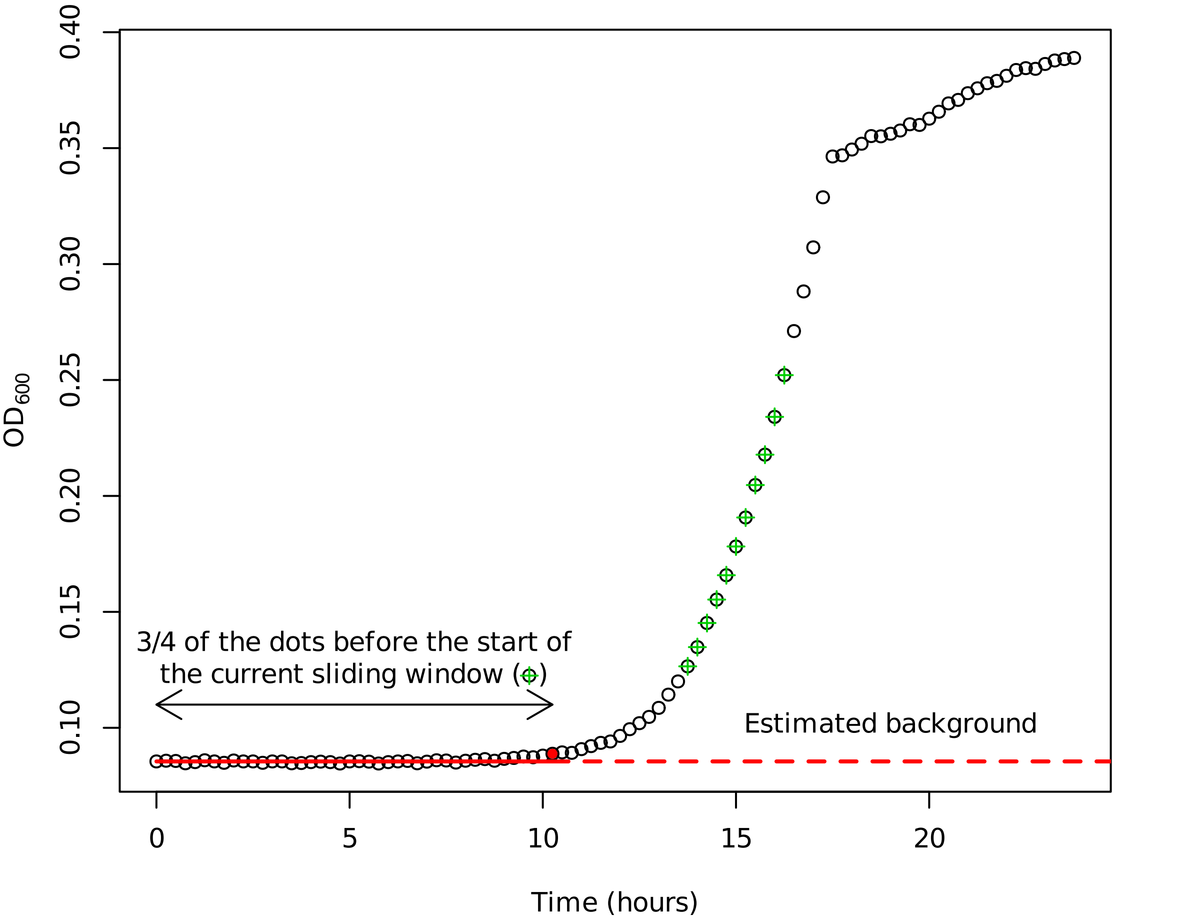

OD600
0.10
0.15
0.20
0.25
0.30
0.35
0.40
0
5
10
15
20
Time (hours)
3/4 of the dots before the start of
the current sliding window ( )
Estimated background
