# Supplementary material for: Evolution of Bacterial Persistence to Antibiotics during a 50,000-Generation Experiment in an Antibiotic-Free Environment
Source: Antibiotics (Basel). 2022 Mar 27;11(4):451. doi: 10.3390/antibiotics11040451 (PMC9028194; doi:10.3390/antibiotics11040451)
Supplement: Supplementary file 1 [file antibiotics-11-00451-s001.zip › antibiotics-1634163-supplementary/Supplementary material 1- Heuristic detection of the exponential growth phase.zip!/Supplementary material 1- Heuristic detection of the exponential growth phase/heterogeneity between initial OD.pdf]

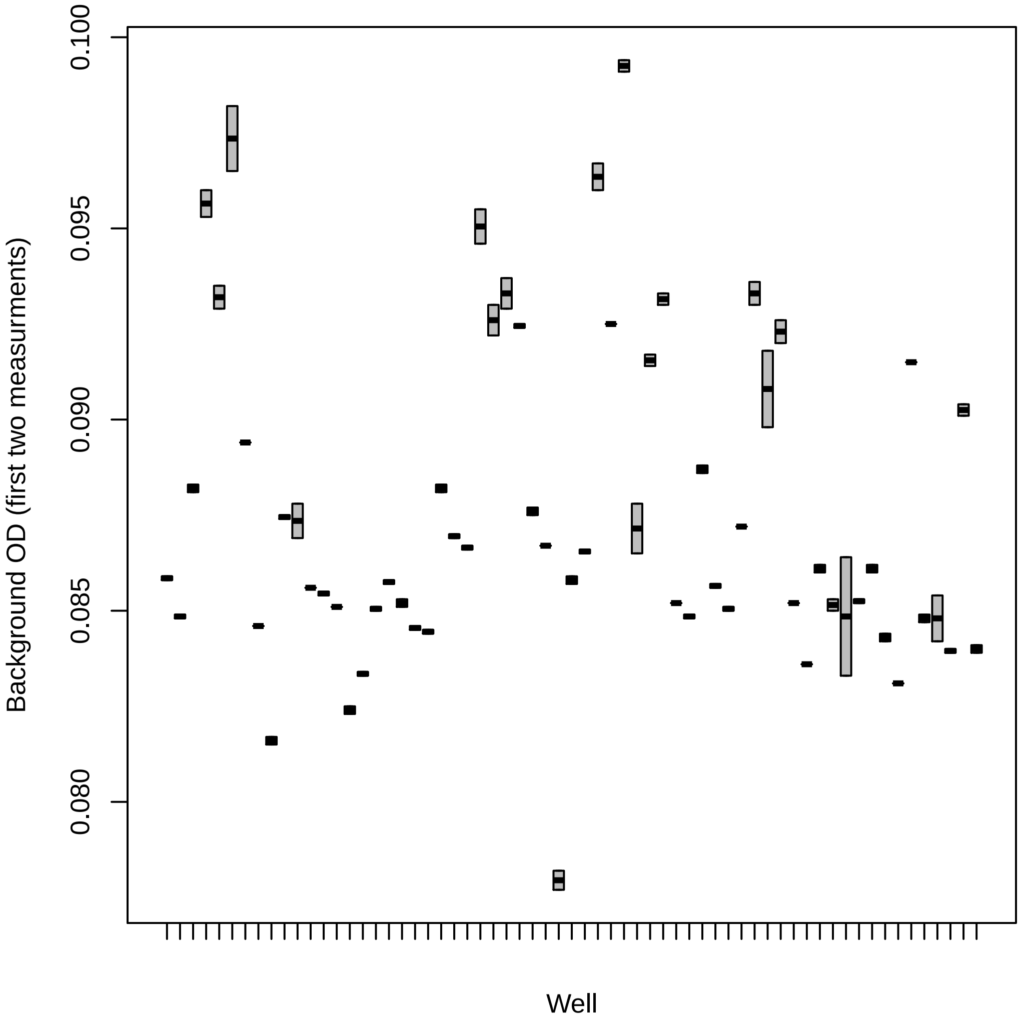
0.100
0.095
0.090
0.085
0.080
Background OD (first two measurments)
Well
